# Overdose prevention activities led by local public health departments, 2019–2023

**DOI:** 10.1186/s13011-024-00612-y

**Published:** 2024-06-04

**Authors:** April Wisdom, Stephanie Haddad, Madhumita Govindu, Francis Higgins, Nikki Filion, Kate Sullivan, Cherie Rooks-Peck

**Affiliations:** 1 Centers for Disease Control and Prevention, National Center for Injury Prevention and Control, Division of Overdose Prevention, 4770 Buford Highway NE, MS S106-8, Chamblee, GA 30341-3717 USA; 2 National Association of City and County Health Officials, Washington, D.C, USA; 3 Joselyn Leavy and Associates, New York, USA; 4 New York City Department of Health and Mental Hygiene, New York, USA

**Keywords:** Opioid, Overdose, Prevention, Treatment, Recovery, Harm reduction, Local health department

## Abstract

**Background:**

Drug overdose deaths in the United States increased to historic levels in recent years, with provisional estimates indicating more than 111,000 deaths in the 12 months ending July 2023. In 2019, the Centers for Disease Control and Prevention’s Division of Overdose Prevention in collaboration with the National Association of City and County Health Officials, funded local health departments (LHDs) to work on overdose prevention activities. This paper aims to: 1) describe the overdose prevention activities that LHDs implemented during the four eighteen-month funding cycles; 2) identify programmatic successes and areas of opportunity for LHDs to consider when implementing future overdose prevention activities; and to 3) inform policy considerations and future overdose prevention programming at the local level.

**Methods:**

We used programmatic data to identify overdose prevention activities implemented by 45 LHDs. Activities were double-coded according to the social-ecological model and the U.S. Department of Health and Human Services Overdose Prevention Strategies and Guiding Principles. We analyzed final codes to identify distribution and overlap of the Strategies and Guiding Principles across the social ecological model co-occurrences.

**Results:**

Approximately 55.9% (*n*=123) of the 220 overdose prevention activities that were coded took place at the community level, 32.3% (*n*=71) at the individual level, 8.6% (*n*=19) at the relationship level, and 3.2% (*n*=7) at the policy level. Most of the activities were coded as coordination, collaboration, and integration (*n*=52, 23.6%), harm reduction (*n*=51, 23.1%), data and evidence (*n*=47, 21.4%) or reducing stigma (*n*=24, 10.9%). Few activities were related to primary prevention (*n*=14, 6.4%), equity (n=14, 6.4%), recovery support (*n*=11, 5.0%), and evidence-based treatment (*n*=7, 3.2%).

**Conclusions:**

Localities have primarily implemented activities focused on the community and individual levels, with most of these centered around coordination, collaboration, and integration; harm reduction; or data and evidence. This study identified gaps in overdose prevention for LHDs related to treatment and health equity and that more interventions should be implemented at the relationship and policy levels. Continuing these efforts is important as LHDs explore opportunities to enhance and expand their work in various strategy areas across the social ecology. Findings from this study may be used to inform localities as they design and implement future overdose prevention activities.

## Background

U.S. drug overdose deaths increased to historic levels in recent years, with provisional estimates indicating more than 111,000 deaths in the 12 months ending October 2023 [1], most of which involved a high-potency opioid (i.e., illicitly made fentanyl and fentanyl analogs) from the illicit drug supply [1]. This dynamic, evolving, and complex problem requires solutions that are tailored and culturally relevant for cities and counties. The solutions include public health acting as the convener for multi-sector collaboration overdose surveillance, harm reduction services, low barrier access to all services and recovery support for people who use drugs (PWUDs). Federal funds have been used to support cities and counties with high overdose burdens to build a strong foundation of cooperation, partnership, and infrastructure across public health, behavioral health, health systems, community organizations, and public safety. This paper describes a federally funded 4-year effort to assist cities and counties with activities that enhance overdose prevention and response infrastructure, support cohesive programs, and address health inequities in overdose.

The Centers for Disease Control and Prevention (CDC)’s Division of Overdose Prevention, in collaboration with the National Association of City and County Health Officials (NACCHO), funded local health departments (LHD) to work on overdose prevention and response-related activities. This collaborative effort, Implementing Overdose Prevention Strategies at the Local Level (IOPSLL), has funded 45 LHDs or their fiscal representatives since 2019 in cities/counties with some of the highest overdose burdens in the country. LHDs were able to be funded during multiple cohorts.

In 2020, the Office of the Assistant Secretary for Planning and Evaluation convened a workgroup of U.S. Department of Health and Human Services (HHS) experts in overdose prevention and substance use disorders and developed the HHS Overdose Prevention Strategy [2]. Based on the four pillars of primary prevention, harm reduction, evidence-based treatment, and recovery support, the strategy incorporates principles related to enhancing equity, using data and evidence to guide actions, improving coordination, collaboration, and integration across multiple sectors, and reducing stigma. Each HHS Strategy and Guiding Principle can be implemented at multiple levels of the social-ecological model (SEM) to develop a more holistic approach to address overdose.

The SEM was developed to understand how various risk and protective factors contribute to childhood adversity. It describes the individual, relationship, community, and policy factors that increase adversity and can be used to assess how prevention strategies can affect short- and long-term health outcomes [3, 4]. Risk factors for overdose are found at multiple levels of the SEM and include physical and mental health, social connections, access to healthcare, and treatment for substance use disorder [5].

To the best of our knowledge there currently is no published literature that summarizes the overdose prevention strategies and activities that have been implemented by LHDs and their partners on the ground. As federal agencies continue to fund localities to conduct overdose prevention activities, an understanding of the strengths and gaps is needed to identify how LHDs can provide more holistic and comprehensive programming. The current paper summarizes programmatic activities of IOPSLL funding recipients and examines implementation gaps and areas of opportunity. Programmatic activities are categorized by the SEM-level of implementation and the HHS Strategy and Guiding Principles. The results of this study will inform policy considerations and future overdose prevention programming at the local level.

## Methods

**Fig. 1:**
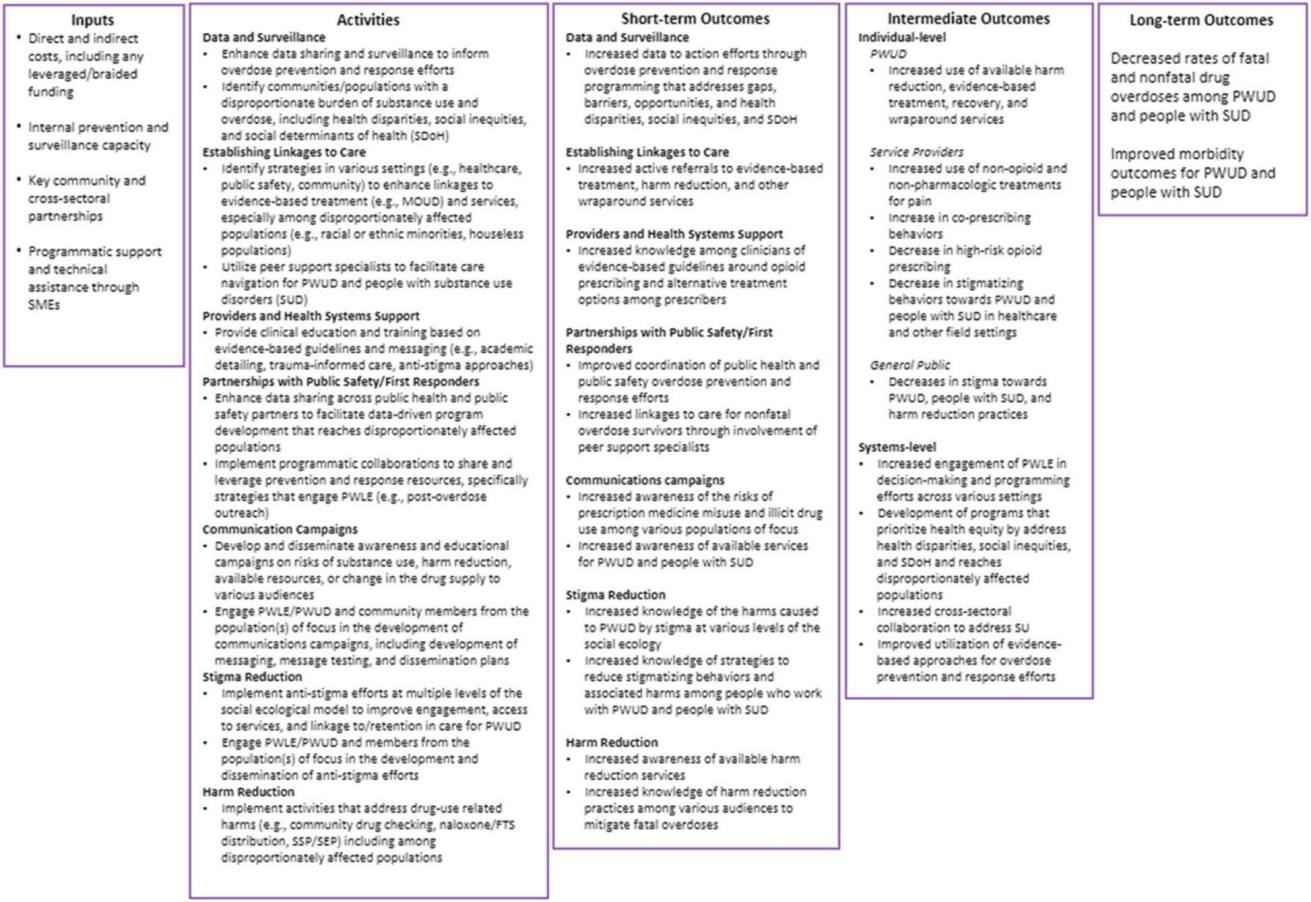
Logic model for implementing overdose response strategies at the local level and outline of overdose prevention strategies at the local level and outline of overdose prevention strategies used by local health departments and their desired outcomes

In 2019, NACCHO, with support from CDC Cooperative Agreement 5 NU38OT000306-03-00 titled *Strengthening Public Health Systems and Services through National Partnerships to Improve and Protect the Nation’s Health*began accepting applications for IOPSLL. Each applicant was funded for 18 months, and since 2019, four cohorts with a total of 45 LHDs have been funded to conduct overdose prevention and surveillance activities. The LHDs were required to implement overdose prevention and response strategies with the greatest impact on the overdose burden [6, 7], which are also reflected in the HHS Prevention Strategies Guiding Principles. Each IOPSLL cohort had different funding requirements. The strategy areas required for funding included surveillance and data sharing, linkages to care, provider and health systems support, partnerships with public safety and first responders, communication campaigns, stigma reduction, health equity, and harm reduction. Table 1 provides details regarding the different IOPSLL funding cycles and the required and optional overdose prevention activities of each funding opportunity. Each of the 45 recipients received between $190,000 and $750,000 per year as well as support from a suite of technical assistance (TA) providers. Table 1 also shows the average funds received per year for each cohort. These providers included members of a high-capacity public health department and programmatic support from CDC and NACCHO staff, as well as experts in data, surveillance, and evaluation. Two jurisdictions (Milwaukee, WI and Seattle and King County, WA) were funded during two different cohorts. See Fig. 1 for the IOPSLL program logic model.

**Table 1:** Description of the Implementing Overdose Prevention Programs at the Local Level (IOPSLL) required and optional strategies and number of sites by cohort

**Cohort (years)**	**Number of sites**	**Average IOPSLL funds awarded per year**	**IOPSLL Strategies in RFA**
Cohort 1 (Dec 2019-Jul 2021)	4	$749,887.33	Establishing Linkages to Care Provider and Health Systems Support Partnerships with Public Safety and First Responders Empowering Individuals to Make Safer Choices Prevention Innovation Projects
Cohort 2 (Dec 2020-Jul 2022)	8	$283,975.57	Establishing Linkages to Care Provider and Health Systems Support Partnerships with Public Safety and First Responders Empowering Individuals to Make Safer Choices Enhanced Surveillance and Data Sharing Prevention Innovation Projects
Cohort 3 (Dec 2021-Jul 2023)	14	$295,724.09	Establishing Linkages to Care Provider and Health Systems Support Partnerships with Public Safety and First Responders Communications Campaigns^a^ Harm Reduction Activities^a^ Enhanced Surveillance and Data Sharing Innovation Prevention Projects
Cohort 4 (Nov 2022-Jan 2024)	21	$190,357.20	Establishing Linkages to Care Provider and Health Systems Support Partnerships with Public Safety and First Responders Communications Campaigns Harm Reduction Activities *Surveillance and Data Sharing [REQUIRED]*^b^ Stigma Reduction ^c^ *Health Equity [REQUIRED]*^d^

^a^ The Empowering Individuals strategy was changed and split into two key components for Cohort 3: Communications Campaigns and Harm Reduction Activities

^b^ Surveillance and Data Sharing became a required strategy for all sites for Cohort 4. To be eligible for funding, sites were required to propose activities to create or enhance the collection of timely fatal and/or non-fatal overdose data and/or EMS data and include efforts to identify populations and communities disproportionately affected by substance use and overdose. Applicants with existing surveillance infrastructure were required to propose activities to improve data linkage and/or data reporting and dissemination, how data would be used inform their prevention efforts, including addressing health disparities, social inequities, and other social determinants of health

^c^ Stigma Reduction was added as a standalone strategy for Cohort 4

^d^ Health Equity was not a standalone strategy for Cohort 4, but it was required that funded jurisdiction embed health equity components into all of their overdose prevention activities funded under the grant

### Activity inventory

Each funded LHD developed a workplan that guided their overdose prevention efforts throughout the funding period. Each site’s workplan implemented between two and fifteen (mean number of activities=4.9 per funded site) that were directly linked to the required strategy areas of the funding opportunity and were comprised of multiple objectives. Workplan activities (*n*=222) were extracted into an Excel spreadsheet to describe the LHD’s overdose prevention activities. The spreadsheet included information about strategies, activities, objectives, and population of focus. Notes from monthly calls with the LHDs were used to add additional context to activities. A total of 220 workplan activities were coded. Two activities were removed because the LHDs did not implement them while they were receiving IOPSLL funding.

### Coding

Workplan activities were extracted and coded according to the SEM level (i.e., individual, relationship, community, and policy), HHS Overdose Prevention Strategy (i.e., primary prevention, harm reduction, evidence-based treatment, and recovery support), and guiding principles (i.e., equity; data and evidence; coordination, collaboration, and integration; and reducing stigma). A codebook was developed with key definitions and examples for each code and was provided to the coding team which consisted of six persons (initials: AW, SH, MG, FH, NF, KS). The SEM and HHS Overdose Prevention Strategies and Guiding Principles were used to develop the codebook [2, 4]. For the SEM, an activity was coded at the individual level if it directly benefitted the population of focus. The relationship level referred to activities that directly benefitted persons closely connected to those at risk of overdose such as family, friends, first responders, and healthcare providers. An activity was coded at the community level when the focus was on the larger community in which PWUDs resided such as schools, neighborhoods, and libraries. Lastly, the policy level referred to activities that focused on supporting or changing social norms or policies that influence overdose prevention and response.

One member of the team compared all coding responses and identified discrepancies (*n*=79). Three members of the coding team met to discuss the workplan activities and to reconcile the coding discrepancies until a consensus was reached. Coding occurred between October 2022 to July 2023.

### Analysis

Final codes were uploaded into R version 4.3.1 (2023-06-16 ucrt) for analysis to identify the distribution of the codes and the co-occurrence of SEM level and HHS Overdose Prevention Strategies and Guiding Principles.

## Results

Table 2 describes the activities that were coded for each LHD. Most activities among IOPSLL recipients occurred at the community level of the SEM and under the Coordination, Collaboration, and Integration guiding principle.

**Table 2:** Activities that have been funded and implemented during the four years of the Implementing Overdose Prevention Programs at the Local Level (IOPSLL) program.

**Jurisdiction**	**Cohort**	**IOPSLL Activity Description**	**SEM Level**	**HHS Strategy**
Austin, TX	4	Expand outreach and education for overdose prevention to bars, clubs and other businesses serving young adults ages 18-25 years	Community	Harm Reduction
		Expand education and outreach for overdose prevention for individuals who are formerly incarcerated	Individual	Coordination Collaboration and Integration
		Conduct outreach and educational presentations on overdose prevention with staff of community-based organizations in reentry programs	Community	Recovery Support
		Provide linkage to care services to connect individuals who are formerly incarcerated with social determinants of health resources	Individual	Recovery Support
		Produce and distribute Quarterly Overdose Surveillance Report and Dashboard to inform prevention efforts	Community	Data and Evidence
		Increase awareness about opioid prescribing and SUD	Community	Primary Prevention
		Increase awareness of fentanyl in the drug supply and availability of naloxone in community at greater risk of overdose	Community	Primary Prevention
Baltimore, MD	3	Purchase and identify locations for public health vending machines and syringe drop-box equipment	Community	Harm Reduction
		Develop and deploy educational items for distribution to increase knowledge and awareness of the new resources	Individual	Harm Reduction
		Implement anti-stigma media campaign to educate the community about SUD and available resources	Community	Reducing Stigma
Bristol County, MA	3	Increase the scalability of local media campaign website about substance use to reach the entire county	Community	Primary Prevention
		Conduct outreach to connect people with treatment and supportive services and distribute harm reduction supplies and information	Individual	Harm Reduction
		Conduct immediate outreach to overdose survivors and disseminate naloxone, harm reduction supplies, and information	Individual	Coordination Collaboration and Integration
Camden County, NJ	2	Secure subcontractors to provide harm reduction specialists and/or certified peer recovery specialists to be placed in homeless shelters to refer individuals to MOUD	Individual	Evidence-Based Treatment
		Secure harm reduction specialists/certified peer recovery specialists to link unstably housed community members to harm reduction services	Individual	Harm Reduction
		Secure harm reduction specialists/certified peer recovery specialists to link unstably housed community members to recovery services	Individual	Recovery Support
Elizabeth, NJ	4	Create follow up resources to support recovery initiatives for people with SUD seeking recovery and hire a community outreach worker	Individual	Reducing Stigma
		Implement a state-developed media campaign to educate first responders and family members to use appropriate language to promote successful recovery	Relationship	Reducing Stigma
		Develop a more intensive information delivery system focused on individuals and family members of individuals in recovery	Community	Reducing Stigma
		Use data and surveillance dashboards to monitor and respond to overdose spikes	Community	Data and Evidence
		Create a data collection method to develop a matrix for reviewing program outcomes	Community	Data and Evidence
		Provide harm reduction supplies to reduce fatal overdoses and other health issues related to substance use	Individual	Harm Reduction
Manchester, NH	4	Develop an overdose response team to respond in real time via community outreach and distribute harm reduction supplies to PWUD and community partners	Community	Harm Reduction
		Conduct early morning and late-night outreach to people at greatest risk of overdose including linkages to treatment for SUD if desired	Individual	Evidence-Based Treatment
		Ongoing outreach with local police and public health and safety team to contact individuals and offer support and connection to services, including harm reduction supplies	Individual	Harm Reduction
		Utilize daily syndromic surveillance, reporting and analysis to improve data sharing and surveillance to inform overdose prevention and response efforts with community partners	Community	Data and Evidence
		Train community members and partners to better support PWUD by applying harm reduction modalities and principles and to better understand health equity issues	Community	Harm Reduction
		Develop an overdose prevention plan with recommendations for action	Policy	Primary Prevention
		Conduct early morning and late-night outreach to people at greatest risk of overdose and distribute harm reduction supplies	Individual	Harm Reduction
Dayton & Montgomery County, OH	2	Hire and train four peer recovery supporters that will respond to drug overdose survivors in the emergency departments	Individual	Coordination Collaboration and Integration
		Implement a message system to include overdose spike alerts, mental health and SUD treatment options, and naloxone training	Individual	Data and Evidence
Denver, CO	3	Hire a data analyst to acquire data, clean and analyze data, and report findings	Community	Data and Evidence
Forsyth County, NC	3	Provide harm reduction supplies to PWUD and their loved ones	Individual	Harm Reduction
		Provide naloxone education and training and education on wound care and safer drug use to individuals who use drugs and their loved ones	Individual	Harm Reduction
		Create media campaign(s) to educate residents about principles and practices of harm reduction	Community	Harm Reduction
Greene County, OH	4	Conduct weekly mobile outreach and expand services to reach underserved and BIPOC communities	Individual	Harm Reduction
		Develop an anti-stigma workshop to increase public serving positions' ability to reduce stigma against PWUD in three jurisdictions	Policy	Reducing Stigma
		Effectively identify system gaps and opportunities for innovative community-specific overdose prevention and intervention strategies through quarterly review of countywide overdoses	Community	Data and Evidence
		Expand the quick response team into an additional high overdose area(s)	Community	Coordination Collaboration and Integration
		Increase access to naloxone through the implementation of free naloxone kiosks with focus on high-burden areas	Community	Harm Reduction
Hampden County, MA	3	Increase awareness of SUD resources and safer drug use practices for people with SUD	Individual	Harm Reduction
		Increase access to naloxone for people with SUD and providers through training community members on naloxone administration	Relationship	Harm Reduction
		Increase collaboration of outreach recovery coaches, harm reduction, and healthcare providers	Community	Coordination Collaboration and Integration
		Implement several media campaigns to decrease discrimination, prejudice, and stigma towards people with SUD among residents and providers	Community	Reducing Stigma
		Build an improved data infrastructure to better understand overdose trends and increase outreach and referrals	Community	Data and Evidence
Hampton Health District, VA	4	Expand care coordination network for providers and substance users through the software platform	Individual	Coordination Collaboration and Integration
		Increase education and training on trauma-informed care among community partners	Community	Equity
		Increase education and training on health equity in the overdose response among community partners	Community	Equity
		Increase education and training on stigma for media partners	Community	Reducing Stigma
		Create website and media campaign and host outreach events to increase community awareness of health district-led substance use program, prevention programs, and harm reduction initiatives and policies	Community	Primary Prevention
		Standardize data metrics, evaluation, and analytics across community partner groups	Community	Data and Evidence
		Incorporate and build out health equity across all aspects of health district-led substance use program and community partners through a health equity survey	Community	Equity
Hennepin County, MN	4	Expand and enhance our current services to establish care coordination and wrap-around services for clinic clients utilizing harm reduction services, and for those taking MOUD	Individual	Coordination Collaboration and Integration
		Expand and enhance harm reduction services to Native American Community by providing harm reduction supplies during outreach	Individual	Harm Reduction
		Expand and enhance data collection and analysis by initiating qualitative and quantitative data collection and organization to identify gaps in service	Community	Data and Evidence
Jefferson County, AL	2	Implement 24/7 peer coverage in emergency departments with on-call or embedded peer services	Individual	Coordination Collaboration and Integration
		Integrate alerts into emergency departments electronic health record (EHR) to encourage consultation of peers and dispensing of naloxone to patients presenting with drug overdose	Policy	Coordination Collaboration and Integration
		Provide educational material to promote the use of peers by healthcare providers	Community	Coordination Collaboration and Integration
		Identify harm reduction and anti-stigma materials through peer relationships in participating hospitals	Community	Harm Reduction
		Distribution harm reduction and anti-stigma materials to providers in participating hospitals through peer relationships	Relationship	Harm Reduction
Jefferson County, MO	4	Develop and implement data sharing via a cost-effective data extraction and transformation system among county and region	Community	Data and Evidence
		Develop and implement a robust, user-friendly community data dashboard	Community	Data and Evidence
		Increase capacity of community to tailor interventions to reduce substance use and overdose related morbidity, mortality or associated harm through data	Community	Data and Evidence
		Conduct focus group discussions with individuals at greater risk of overdose about causes of substance use and launch a media campaign to engage the general population and encourage the use of the overdose data dashboard	Community	Data and Evidence
Knox County, TN	3	Create a formal recurrence prevention curriculum for distribution to other agencies	Community	Recovery Support
		Implement a minority syndemic outreach plan to provide education on syndemic-related topics, rapid testing for human immunodeficiency virus (HIV) and Hepatitis C (HCV), and naloxone distribution by partner agencies	Individual	Equity
		Develop and deploy community anti-stigma media campaign with special attention to justice systems staff, medical providers, and influencers in the community	Community	Recovery Support
		Hire and train a harm reduction navigator to provide linkage to care	Individual	Coordination Collaboration and Integration
		Create and disseminate a toolkit for other local health departments to start their own harm reduction navigation programs	Community	Harm Reduction
		Establish a post overdose response team to conduct outreach to individuals who have experienced a nonfatal overdose and did not go to an emergency department or hospital	Individual	Coordination Collaboration and Integration
		Establish a contract with a first responder agency to input data on nonfatal overdoses into Overdose Mapping and Application Program (ODMAP)	Community	Data and Evidence
		Assist individuals in recovery with accessing recovery housing through a recovery housing support fund	Individual	Recovery Support
Ledge Light Health District, CT	4	Enhance information exchange and team connectedness of local harm reduction alliance through more robust and timely systems	Community	Data and Evidence
		Improve information exchange and connectedness between local harm reduction alliance and PWUD	Relationship	Coordination Collaboration and Integration
		Improve linkages to care and awareness of overdose prevention and response education for people whose primary language is Spanish	Individual	Equity
		Improve linkages to care and awareness of overdose prevention and response education for members of the Tribal Nations in jurisdiction	Individual	Equity
		Develop materials and information about accessing evidence-based care for PWUD who read languages other than English to improve knowledge of available services	Individual	Equity
		Develop multi-media campaign to increase knowledge of harm reduction and overdose prevention with general audience and community partners	Community	Harm Reduction
Lorain County, OH	2	Partner with Sheriff’s office to establish linkages to care to reduce the burden of overdose in the community by distributing 400 naloxone kits to high-risk inmates or inmates who may be in a position to respond to a suspected drug overdose upon release	Individual	Harm Reduction
		Create and implement a data tracking process to monitor program activities and support program improvement to reduce the burden of overdose in the community by distribution of 400 naloxone kits to high-risk inmates or inmates who may be in a position to respond to a suspected drug overdose upon release	Community	Data and Evidence
		Develop and implement a mass-market communications campaign to share evidence-based messaging about drug use and people who use drugs and address stigma around drug use	Community	Reducing Stigma
Los Angeles, CA	1	Implement MOUD media campaign to increase awareness of services	Community	Data and Evidence
		Develop and implement SUD resource guide and mobile application to increase awareness and referral coordination to services	Individual	Coordination Collaboration and Integration
Lucas County, OH	3	Implement academic detailing to increase evidence-based harm reduction practices amongst healthcare providers in hospital emergency department settings	Community	Harm Reduction
		Develop and implement media campaigns to share evidence-based messaging about substance use and PWUD that address stigma about substance use	Relationship	Reducing Stigma
		Incorporate peer support within overdose prevention strategies to establish better linkages to care	Individual	Coordination Collaboration and Integration
		Enhance data sharing across public health, mental health and public safety partners to leverage prevention and response resources	Community	Data and Evidence
Marion County, IN	1	Educate medical providers on opioid prescribing and non-opioid pain management options	Community	Primary Prevention
		Conduct anti-stigma media campaign for general public and assess public's awareness, knowledge, and attitudes towards SUD and naloxone use	Community	Reducing Stigma
		Improve inter-agency coordination and linkage to care	Community	Coordination Collaboration and Integration
Mercer County, NJ	4	Use syndromic surveillance to identify emerging overdose trends and surges collaboratively with local emergency responders and develop a notification system	Community	Data and Evidence
		Develop a media campaign that brings awareness and health education on naloxone, fentanyl, and the emerging public health crises around xylazine	Community	Harm Reduction
		Lease two dispensing kiosks that will provide 24 hrs. of low-barrier access to harm reduction supplies	Individual	Harm Reduction
Meriden Health and Human Services, CT	4	Distribute harm reduction supplies at community events and presentations to reach people not referred to/involved in local EMS led naloxone and medical services referral program	Community	Harm Reduction
		Provide anti-stigma training and resources to emergency department providers and those who work with PWUD or people with SUD	Relationship	Reducing Stigma
		Surveil evolving overdose trends and create a data dashboard on the city website, sharing data in a way that is meaningful and accessible to the general public and community partners	Community	Data and Evidence
		Implement an anti-stigma media campaign for the general public	Community	Reducing Stigma
Milwaukee, WI	1	Provide harm reduction, treatment, and SUD resources in local park for unhoused and SUD populations	Individual	Coordination Collaboration and Integration
		Identify and attempt to reach overdose survivors and offer resources for ongoing care of the overdose survivor and their loved ones	Individual	Coordination Collaboration and Integration
		Conduct SUD awareness, motivational interviewing, and trauma informed care trainings to first responders	Community	Reducing Stigma
		Create Palm card for SUD resources	Individual	Coordination Collaboration and Integration
		Develop overdose surveillance database	Community	Data and Evidence
		Review overdose deaths to identify hot spots for opioid, fentanyl, or other lethal combinations of substances	Community	Data and Evidence
		Develop monthly report of all fatal and non-fatal overdoses	Community	Data and Evidence
	3	Distribute naloxone to overdose survivors	Individual	Harm Reduction
		Identify and reach out to patients who have experienced a nonfatal overdose	Individual	Coordination Collaboration and Integration
		Provide educational resources to overdose survivors on harm-reduction methods, treatment, and other community resources	Individual	Coordination Collaboration and Integration
		Provide educational resources to families of overdose survivors on harm-reduction methods, treatment, and other community resources	Relationship	Coordination Collaboration and Integration
		Link overdose survivors to treatment	Individual	Coordination Collaboration and Integration
		Link overdose survivors to recovery services	Individual	Coordination Collaboration and Integration
		Monthly reports on patient interaction with community paramedics and access to treatment and recovery services	Community	Coordination Collaboration and Integration
		Report real-time overdose data of all fatal and non-fatal overdoses	Community	Data and Evidence
Nashville & Davidson County, TN	3	Deploy health educators to engage with community partners and distribute materials to increase the number of community members who can support overdose and SUD related issues in prioritized communities	Relationship	Coordination Collaboration and Integration
		Develop a robust and enhanced qualitative data/information system focusing on disproportionately affected Coptic and African American communities	Community	Data and Evidence
		Analyze data to increase ability to adapt overdose response programs, across diverse communities, to respond to changes in SUD and overdose trends	Community	Equity
New Haven, CT	4	Create a public-facing data sharing platform	Community	Data and Evidence
		Create a county-wide data sharing initiative with local health departments, first responders, and outreach teams	Community	Data and Evidence
		Train providers and pharmacists on safe opioid prescribing	Relationship	Primary Prevention
		Train community members on stigma, harm reduction, and overdose prevention	Community	Equity
		Develop a media campaign on overdose prevention focused on city residents	Community	Reducing Stigma
North Central District, CT	4	Enhance and increase the level of data sharing within health district member towns, town health department, local health district, prevention coalitions, and first responders	Community	Data and Evidence
		Increase engagement with first responders to identify hot spots and identify locations for overdose prevention and naloxone training	Community	Data and Evidence
		Implement a media and outreach campaign to increase the number people with SUD and those close that are connected to recovery, case management and treatment services	Individual	Evidence-Based Treatment
		Work with local harm reduction alliance to reach all local health district member-towns and the towns served by neighboring health district with mobile harm reduction services	Community	Evidence-Based Treatment
		Work with local harm reduction alliance to reach all local health district member-towns and the towns served by neighboring health district with mobile harm reduction services to increase the number of treatment referrals for SUD	Individual	Evidence-Based Treatment
Ocean County, NJ	2	Secure an agency to develop an awareness and educational campaign for general overdose information and information specific to overdose spikes	Community	Primary Prevention
		Develop an overdose response plan with state police and local partners to streamline ODMAP, surveillance, and other local data collection to inform community outreach and the development of a media campaign	Community	Data and Evidence
Oneida County, NY	3	Provide harm reduction and trauma-informed care trainings opportunities for task force partners	Community	Harm Reduction
		Coordinate multi-agency, street engagement team outreach to provide on-demand access to MOUD, harm reduction and wrap around services to people in identified high burden neighborhoods	Individual	Coordination Collaboration and Integration
		Develop an anti-stigma media campaign targeted at various audiences	Community	Reducing Stigma
Onondaga County, NY	3	Conduct community outreach to increase harm reduction activities	Individual	Harm Reduction
		Implement stigma reduction and naloxone distribution media campaign to increase awareness of overdose epidemic and promote naloxone distribution and use in prioritized zip codes	Community	Reducing Stigma
		Implement management supports to assist highest need populations with SUD by referring individuals to services	Individual	Coordination Collaboration and Integration
		Implement a data sharing process to increase community understanding of overdose/substance use trends and in turn increase community capacity to respond/mitigate/prevent SUD or fatal overdoses	Community	Data and Evidence
		Expand PAX Good Behavior Game in local school districts to increase children’s resiliency and in the future reduce rates of substance use and fatal overdoses	Community	Primary Prevention
Orange County, FL	4	Create a public health and safety team that blends input from public health, public safety, providers, and the community to facilitate a deeper understanding of the missed opportunities for intervention that may prevent overdose deaths	Individual	Coordination Collaboration and Integration
		Identification of individuals with repeat overdoses and provide linkages to physical and behavioral health care, including transportation and cell phones	Individual	Coordination Collaboration and Integration
		Enhance community outreach programs for naloxone training and distribution, bystander training on overdose recognition and response, stigma reduction messaging among businesses with multiple EMS naloxone reversals, college students, and other community members	Community	Harm Reduction
Pima County, AZ	3	Distribute fentanyl test strips to local agencies, partners, and coalitions for use in clinical settings or directly to PWUD	Community	Harm Reduction
		Provide presentations to local law enforcement and begin distributing a standardized presentation for providers, jails, and partners who work with people with SUD in order to bolster community harm reduction	Community	Coordination Collaboration and Integration
		Develop a media campaign and distribute communication and educational materials to reduce perpetuation of stigma and negative stereotypes around substance use towards communities of color that have been historically marginalized	Community	Reducing Stigma
		Develop and implement “safe space” concept to outpatient clinics that will provide harm reduction and resource distribution for people with substance use	Individual	Harm Reduction
		Develop and implement “safe space” concept to outpatient clinics that will provide linkage to care and resource distribution for people with substance use	Individual	Coordination Collaboration and Integration
		Expand peer navigation services in community-based organizations in priority communities (i.e., Native Americans, incarcerated individuals, patients at hospitals/emergency departments) and provide overdose prevention services and support	Individual	Coordination Collaboration and Integration
Portland, ME	4	Create a county level overdose alliance to increase resource and sharing, identify needs and gaps, and support community-driven evidence-based interventions	Community	Data and Evidence
			Community	Coordination Collaboration and Integration
		Increase data collection and sharing tools and processes throughout county to identify gaps and drive training efforts and allocation of resources	Community	Data and Evidence
		Expand outreach efforts to PWUD to organizations and agencies throughout county and partner with organizations to expand the distribution of harm reduction supplies	Relationship	Harm Reduction
Portsmouth, VA	4	Review, coordinate, and disseminate local data to promote public awareness of the burden and opportunities to prevent overdose	Community	Data and Evidence
		Convene an overdose prevention task group to assist with strategic planning and implementation of overdose prevention efforts	Community	Coordination Collaboration and Integration
		Use a care coordination platform, to link PWUD to care and resources	Individual	Coordination Collaboration and Integration
		Provide anti-stigma trainings and resources for EMS, public safety, public health, and peer support specialists	Relationship	Reducing Stigma
		Use a care coordination platform, to link PWUD to care and resources	Community	Primary Prevention
		Expand post overdose outreach program for individuals who experienced a non-fatal overdose and distribute harm reduction kits	Individual	Harm Reduction
		Expand post overdose outreach program and provide awareness literature for family members and caregivers of those affected	Relationship	Primary Prevention
Richmond & Henrico Health Districts, VA	4	Collect qualitative data from resident-led community conversation to pair with existing overdose and substance use data to increase health district understanding of the drivers of substance use	Community	Equity
		Fund community organizations to provide navigation resources to increase access to supportive services for people with SUD re-entering the community and those pursuing recovery	Individual	Equity
Rio Arriba County, NM	4	Expand distribution of harm reduction resources directly to people at risk of overdose	Individual	Harm Reduction
		Engage with local establishments and bus service to offer naloxone training and install free naloxone kiosks at key locations	Community	Harm Reduction
		Expand existing coalition to increase partner engagement to include first responders, jail-based staff, school counselors, and harm reduction case management/certified peer support workers	Community	Coordination Collaboration and Integration
		Expand child protective social worker based court diversion to work with justice-involved individuals and at greater risk for overdose and expand non-traditional support resources for participants in peer-supported probation	Individual	Recovery Support
		Expand data gathering and analysis and develop a shared public-facing data dashboard for the public and community partners to reduce overdose	Community	Data and Evidence
Rock County, WI	4	Develop and distribute overdose resource kits to PWUDs and/or have experienced an overdose	Individual	Harm Reduction
		Partner with local harm reduction provider to promote naloxone and fentanyl test strips to high burden population	Individual	Harm Reduction
		Increase data sharing through an expanded public-facing dashboard	Community	Data and Evidence
		Implement an overdose alert system in partnership with state level department of health services	Policy	Data and Evidence
		Coordinate opioid use disorder (OUD) anti-stigma trainings for healthcare workers, law enforcement and EMS	Relationship	Reducing Stigma
		Implement overdose awareness media campaign directed at healthcare workers, law enforcement and EMS	Community	Reducing Stigma
San Francisco, CA	3	Implement neighborhood-based testing to allow active substance users to check the quality, purity, and safety of drugs they will potentially use	Individual	Harm Reduction
		Expand the scope and quality of opioid use assessment, response, and treatment linkage services within hospital-based emergency departments	Relationship	Coordination Collaboration and Integration
		Develop and launch an overdose and treatment dashboard for the city and county	Community	Data and Evidence
Seattle & King County, WA	1	Provide x waiver training to prescribers	Relationship	Evidence-Based Treatment
		Develop naloxone co-prescribing protocols	Policy	Harm Reduction
		Implement naloxone co-prescribing protocols	Relationship	Harm Reduction
		Distribute harm reduction supplies to people who inject drugs (PWID)	Individual	Harm Reduction
		Develop and implement media campaign focused on fentanyl risks amongst youth	Individual	Harm Reduction
		Develop and disseminate messaging strategy regarding harm reduction for adults at greater risk of overdose	Individual	Harm Reduction
		Implement media campaign for general public to increase awareness of OUD treatment	Community	Evidence-Based Treatment
		Design and implement an assessment that determines the prevalence of fentanyl in local drug markets	Community	Data and Evidence
	3	Expand existing community-based organization training of trainers for overdose prevention program	Community	Harm Reduction
		Continuation of youth focused media campaign that provides information about fentanyl, confidential naloxone access, overdose prevention resources, and overcoming stigma	Community	Primary Prevention
		Hire post overdose social services specialist and follow-up with non-fatal overdose survivors and families of overdose decedents	Individual	Coordination Collaboration and Integration
Seminole County, FL	2	Strengthen programmatic partnerships and protocols to leverage the resources and expertise of public safety and first responder organizations	Community	Coordination Collaboration and Integration
		Enhance the local response to non-fatal overdose calls by providing community paramedic and SUD therapeutic overlay services in the field	Community	Coordination Collaboration and Integration
		Provide critical personnel to respond to non-fatal overdoses and improve delivery of substance use prevention and intervention services and improve awareness of community resources	Individual	Coordination Collaboration and Integration
		Increase and improve coordination among organizations that provide care or enable linkages to care for individuals that have a SUD	Community	Coordination Collaboration and Integration
		Launch marketing and data sharing efforts to improve community awareness of available SUD services and integrate substance use data in a publicly accessible website	Community	Data and Evidence
Snohomish County, WA	4	Initiate data-sharing contracts with community partners and first responder agencies to improve surveillance of overdose trends among populations disproportionately impacted by overdose	Community	Data and Evidence
		Create and disseminate media campaign materials for a multilingual communications campaign to reach BIPOC populations	Community	Equity
		Update locally hosted website with relevant content and appropriate translations to reach 20,000 community members with a focus on BIPOC communities	Community	Equity
		Implement a naloxone leave-behind program among first responders and community-based partners who serve BIPOC community members	Relationship	Harm Reduction
		Train community members and first responders in trauma-informed care when responding to an overdose	Relationship	Equity
Ulster County, NY	2	Implement a walk-in center to distribute naloxone and fentanyl test strips and provide harm reduction education to individuals at-risk of an overdose	Individual	Harm Reduction
		Implement a street outreach program to provide PWUD/PWID with harm reduction education and supplies and provide linkages to care	Individual	Coordination Collaboration and Integration
		Recruit student leaders to develop and implement a media campaign	Community	Primary Prevention
Wyandotte County, KS	4	Establish peer support team and linkage to care referral relationships with local emergency departments, housing organizations, and first responders	Community	Coordination Collaboration and Integration
		Develop county overdose data dashboard	Community	Data and Evidence
		Develop ongoing relationships via peer team with people who can benefit from local recovery resources	Individual	Recovery Support
		Disseminate a resource list to SUD partner organizations for community support and referral options	Relationship	Coordination Collaboration and Integration
		Create and distribute SUD messaging to the general public that assist and elevate the work of the peer support program	Community	Harm Reduction
Vinton County, OH	4	Implement data sharing with the EMS through a data interface from current application to ODMAP for the general public	Community	Data and Evidence
		Develop a post-overdose response team to respond to overdose survivors and their families and/or support persons to offer resource information, naloxone and comfort items	Individual	Coordination Collaboration and Integration
		Plan and hold an anti-stigma health fair for county residents	Community	Reducing Stigma
		Design and implement an anti-stigma multi-media campaign for residents of all ages in the county using community input	Community	Reducing Stigma
		Install free naloxone kiosks in areas with the highest overdose rates and/or least access to naloxone	Community	Harm Reduction
Volusia County, FL	2	Initiate a countywide educational media campaign on the negative effects of SUD, OUD and recovery	Community	Reducing Stigma
		Expand the Overdose Data to Action (OD2A) marketing campaign in high-burden ZIP codes to include radio segments and department vehicle wraps	Community	Recovery Support
		Expand the OD2A countywide media campaign for white males ages 25-49 to include methods not used in the OD2A campaign	Community	Recovery Support
		Educate county leadership regarding harm reduction strategies to advocate for syringe services and expanded naloxone distribution programs	Policy	Harm Reduction
		Host a conference of behavioral health professionals and community residents and create a community plan for ongoing SUD prevention, recovery support, and treatment services	Community	Coordination Collaboration and Integration
		Host community education opportunities for residents and visitors on SUD and overdose prevention	Community	Primary Prevention
		Host community education opportunities for residents and visitors on SUD and overdose prevention	Community	Recovery Support
		Partner with EMS and fire rescue to establish process for receiving and documenting near real-time data on overdose locations, naloxone use, and emergency transport to identify hot spots	Community	Data and Evidence
		Explore and encourage a pilot program establishing a partnership between fire rescue and peer outreach specialists to join EMS at the site of overdose responses	Community	Coordination Collaboration and Integration
		Hire and train peers to ensure universal coverage in emergency departments	Policy	Coordination Collaboration and Integration
		Employ transporters to ensure MOUD patients have access to treatment	Individual	Coordination Collaboration and Integration
		Contract part-time peer outreach specialists and peer outreach supervisor to implement a post-overdose protocol to link overdose survivors to care	Individual	Coordination Collaboration and Integration
		Provide educational opportunities for healthcare professionals to reduce stigma toward people being treated for overdoses and the value of linkages to care	Community	Reducing Stigma
		Add and maintain data, narrative, and links on existing public facing dashboard	Community	Data and Evidence
		Build and maintain a public facing dashboard that shares substance use data of interest to inform community residents	Community	Data and Evidence

**Fig. 2:**
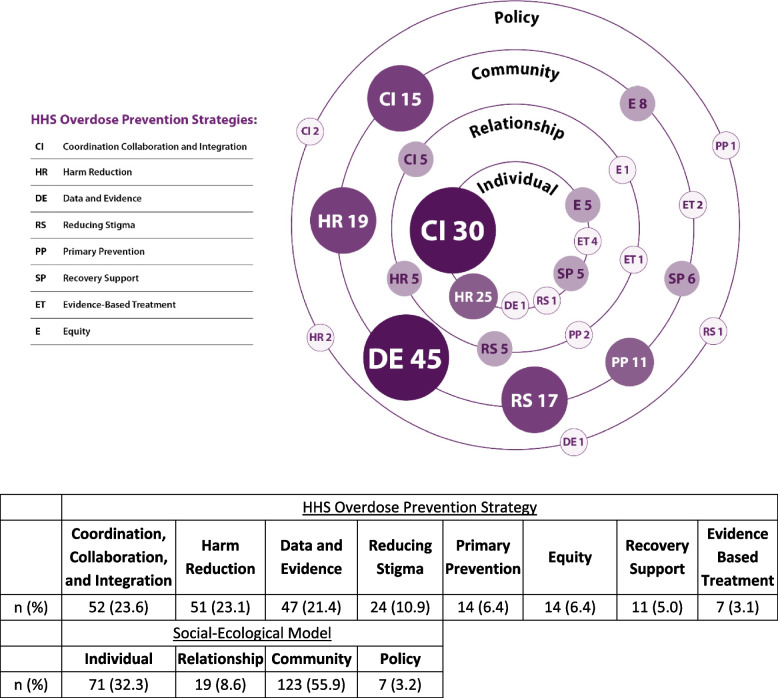
The intersection of Implementing Overdose Prevention Programs at the Local Level (IOPSLL) activities across the Social Ecological Model by HHS Overdose Prevention Strategies and Guiding Principles and the activity numbers and percentages. This figure shows the number of activities being implemented at each level of the social ecological model (SEM) in each of the eight HHS Overdose Prevention Strategies and Guiding Principles across the 45 IOPSLL sites. The HHS Overdose Prevention Strategies and Guiding Principles are represented by abbreviations in the figure and a legend is included. The Strategies and Guiding Principle abbreviations and number of activities are shown in circles of varying size and color intensity as visual indicators of how many activities are being implemented at each SEM level

Figure 2 shows where the activities fell on the levels of the SEM by HHS Overdose Prevention Strategy and Guiding Principle. As seen in Fig. 2, 55.9% (*n*=123) of coded activities occurred at the community level, 32.3% (*n*=71) at the individual level, 8.6% (*n*=19) at the relationship level, and 3.2% (*n*=7) at the policy level. Figure 2 also includes the number and percent of total activities implemented under each SEM level and each HHS Overdose Prevention Strategy and Guiding Principle. Fifty-two activities (23.6%) implemented by the IOPSLL recipients focused on the HHS Guiding Principle of coordination, collaboration, and integration and the majority were implemented at the individual level (57.7%, *n*=30). These activities included linking persons who have experienced a nonfatal overdose to care at emergency departments through emergency medical, paramedic services, post-overdose response programs, and other forms of low-threshold care. The types of care provided included harm reduction materials and other forms of treatment such as medication for opioid use disorder (MOUD) and in-patient services.

Fifty-one activities (23.1%) focused on the HHS Strategy of harm reduction and almost half of those (49.0%, *n*=25) occurred at the individual level. Most of these activities were centered on the distribution of harm reduction materials such as syringes, naloxone, and fentanyl test strips. CDC funds may not be used for purchasing syringes or naloxone and the LHDs braided other funding sources to support this work. Other innovative examples included community drug checking using Fourier transform infrared (FTIR) spectrometers and public health vending machines which are usually stocked with naloxone, testing strips, syringes, and other harm reduction materials.

Forty-seven activities (21.4%) focused on the HHS Guiding Principle of data and evidence, the preponderance (95.7%, *n*=45) of which occurred at the community level. Most used surveillance activities to enhance data sharing and to understand overdose “hotspots” in communities. This knowledge was often used to inform resource distribution, post-overdose response and spike alert systems for partners and providers, and to notify the public about increases in overdoses and/or substance use-related hospitalizations.

Twenty-four (10.9%) activities focused on the HHS Guiding Principle of reducing stigma, most of which (70.8%, *n*=17) occurred at the community level and included media campaigns over billboard and radio. Several campaigns were also comprised of messaging about connection to harm reduction and treatment services.

Fourteen (6.4%) activities focused on the HHS Strategy of primary prevention with much (78.6%, *n*=11) occurring at the community level. Community-level primary prevention activities centered on communication campaigns about overdose risks.

Eleven (5.0%) activities focused on the HHS Strategy of recovery support with most (54.5%, *n*=6) taking place at the community level. These activities included linking people to services such as recovery housing and holding community education opportunities on recovery.

Seven (3.2%) activities focused on the HHS Strategy of evidence-based treatment, and more than half (57.1%, *n*=4) occurred at the individual level. Most activities involved implementing media campaigns and were centered on access to treatment.

Fourteen (6.4%) activities focused on the HHS Guiding Principle of equity, most of which (57.1%, *n*=8) occurred at the community level. Activities were centered on overdose response programs in diverse communities and peer navigation services to high-risk priority communities.

Table 2 describes the partnerships developed or strengthened during the IOPSLL program. Most (*n*=31) IOPSLL-funded LHDs leveraged relationships with public and private entities (*n*=69 unique partners) to provide and implement overdose prevention resources and activities.

Table 3 summarizes the relationship between the SEM level and HHS Overdose Prevention coded IOPSLL activities. Forty-five activities were focused on data and evidence intervention implemented in the community setting, 30 activities revolved around providing linkages to care for individuals, and 25 activities were harm reduction interventions for individuals.

**Table 3:** Contingency table that summarizes the relationship between the Social Ecological Model and HHS Overdose Prevention Strategies and Guiding Principles coded Implementing Overdose Prevention Programs at the Local Level (IOPSLL) activities

		HHS Overdose Prevention Strategies								
		**Coordination, Collaboration, and Integration**	**Data and Evidence**	**Equity**	**Evidence-Based Treatment**	**Harm Reduction**	**Primary Prevention**	**Recovery**	**Reducing Stigma**	**Total n**
Social Ecological Model Level	**Individual**	*n* = 30	*n* = 1	*n* = 5	*n* = 4	*n* = 25	*n* = 0	*n* = 5	*n* = 1	71
		r% = 42.25	r% = 1.41	r% = 7.04	r% = 5.63	r% = 35.21	r% = 0	r% = 7.04	r% = 1.41	
		c% = 57.69	c% = 2.13	c% = 35.71	c% = 57.14	c% = 49.02	c% = 0	c% = 45.45	c% = 4.17	
	**Relationship**	*n* = 5	*n* = 0	*n* = 1	*n* = 1	*n* = 5	*n* = 2	*n* = 0	*n* = 5	19
		r% = 26.32	r% = 0	r% = 5.26	r% = 5.26	r% = 26.32	r% = 10.53	r% = 0	r% = 26.32	
		c% = 9.62	c% = 0	c% = 7.14	c% = 14.29	c% = 9.80	c% = 14.29	c% = 0	c% = 20.83	
	**Community**	*n* = 15	*n* = 45	*n* = 8	*n* = 2	*n* = 19	*n* = 11	*n* = 6	*n* = 17	123
		r% = 12.20	r% = 36.59	r% = 6.50	r% = 1.63	r% =15.45	r% = 8.94	r% = 4.89	r% = 13.82	
		c% = 28.85	c% = 95.74	c% = 57.14	c% = 28.57	c% = 37.25	c% = 78.57	c% = 54.55	c% = 70.83	
	**Policy**	*n* = 2	*n* = 1	*n* = 0	*n* = 0	*n* = 2	*n* = 1	*n* = 0	*n* = 1	7
		r% = 28.57	r% = 14.29	r% = 0	r% = 0	r% = 28.57	r% = 14.29	r% = 0	r% = 14.29	
		c% = 3.85	c% = 2.13	c% = 0	c% = 0	c% = 3.92	c% = 7.14	c% = 0	c% = 4.17	
	**Total n**	52	47	14	7	51	14	11	24	
	n = count; r% = row percentage; c% = column percentage									

## Discussion

This paper describes local level overdose prevention and surveillance activities implemented through NACCHO and CDC’s IOPSLL program. We applied the SEM and HHS Overdose Prevention Strategies and Guiding Principles to showcase how federal-level initiatives may translate at the local level. As seen in Table 2, our study identified that IOPSLL-funded LHDs are implementing programs at multiple levels of the social ecology for people and communities at risk of overdose. Additionally, findings demonstrated that LHDs proposed implementation of programs that would support working at both the community and individual levels and that activities focused on improving community coordination, strengthening surveillance, providing harm reduction services, dispelling stigma, and ensuring that information and services reach key communities disproportionately impacted by overdose. IOPSLL recipients were most likely to engage in overdose prevention activities at the community level of the SEM and under the coordination, collaboration, and integration guiding principle. It is important to note that this is not a complete representation of the overdose prevention response of LHDs as the IOPSLL-funded jurisdictions only reported on their overdose prevention activities that they used the grant dollars to support.

LHDs serve as the hub of public health efforts in their communities. It is important to note that the majority of these LHDs received funding during the COVID-19 pandemic and that the responsibilities of local health departments were impacted during the pandemic. While some IOPSLL-funded activities were delayed due to the pandemic, local health departments and their partners recognized the overdose epidemic did not stop during the pandemic and they persevered while also adapting their activities to minimize the spread of COVID-19. They are well-equipped to collaborate with the many entities that commonly interact with persons affected by or at risk of overdose. This could include convening and leading a community task force to improve communication among local partners or taking a more targeted approach such as working with first responders or hospitals to enhance systems to better tailor services for PWUD. Additionally, a majority of IOPSLL-funded LHDs leveraged relationships with public and private entities to provide these services, such as naloxone training and distribution and care coordination. The LHD’s ability to coordinate efforts allowed them to play a unique and critical role in the overall community response to the overdose epidemic.

Harm reduction is a critical HHS Overdose Prevention Strategy and was commonly administered by IOPSLL recipients. Reducing the risks and harms associated with substance use is critical to keeping persons alive and within reach of services supporting them. In this sphere, IOPSLL recipients implemented numerous activities at both the community and individual levels. Many health departments, in keeping with their role as community collaborators, conducted naloxone trainings or other educational sessions for organizations such as local businesses or first responders. They also served PWUD directly by distributing naloxone and fentanyl test strips and operating or supporting local syringe services programs.

LHDs through IOPSLL implemented stigma reduction activities that mostly occurred at the community level. PWUD often experience stigma from family, service providers, and the greater public which negatively impacts their access to and use of healthcare services [8–10]. To better address stigma, many LHDs implemented communications campaigns such as CDC’s Stop Overdose [11] and others developed by their teams. Stigma reduction messaging was shared through various channels such as social media, radio, billboards, buses, and gas station ads. One jurisdiction partnered with a local peer recovery group to develop a digital and paper campaign that shared the peers’ experience with substance use and recovery. LHDs also implemented activities that focused on stigma reduction among those who are likely to interact and work with PWUD. These activities included training first responders to offer trauma-informed care and trainings for healthcare providers focused on some of the risk factors for developing a substance use disorder (SUD). Many of the LHDs engaged with the community and with people with lived and living experience to tailor their stigma reduction activities. Reducing stigma is essential to support overdose prevention services to facilitate recovery for PWUD.

LHDs recognized the need to support and enhance recovery services and implemented activities to facilitate recovery along with wellness to improve the quality of life for PWUD. These services included strengthening the recovery workforce, as well as enhancing and encouraging the use of recovery support services. LHDs have assisted with and enhanced this support in their communities through IOPSLL by hiring peer support workers to link persons to resources and to connect those in recovery to housing. Many barriers and challenges hinder the availability and uptake of recovery services. They include stigma, residential instability, financial insecurity, confidentiality concerns, and lack of access to treatment services and peer support groups [12–14]. LHDs should address these barriers and continue to assist and strengthen recovery support in their communities.

LHDs engaged in and supported primary prevention efforts through IOPSLL in addition to providing harm reduction, treatment, and recovery services to PWUDs in their communities. LHDs participated in several primary prevention activities with all but three being implemented at the community level. Examples of IOPSLL-supported activities included academic detailing on safe opioid prescribing, non-opioid pain management options among prescribers and pharmacists and implementing overdose prevention communication campaigns for the public. One LHD expanded the reach of the PAX Good Behavior Game [15] to local school districts to reduce substance use and fatal overdoses by enhancing students’ resiliency. Effective prevention strategies are multidisciplinary and should occur at each of the SEM levels [4]; Findings demonstrate the importance of LHDs implementing activities at the individual, relationship, and policy levels in addition to showcasing the strengths of primary prevention at the community level.

The ability to work at both the community and individual levels is an important strength of health departments. Their ability to understand and mobilize community-wide care is key; however, services should ultimately reach those at risk of overdose. The IOPSLL program emphasizes the need to meet persons where they are and continue to distribute resources such as naloxone to those most likely to experience or witness an overdose. This ground-level work is also a vital conduit to further services and offers an opportunity to provide education to PWUD [16]. Continuing to deliver these services will ensure that vital tools are in the hands of those who need them while also deepening the mutual relationship and understanding between the health department and those at risk of overdose.

Study findings presented gaps in services or areas where implementation was low and highlighted HHS Overdose Prevention Strategies and Guiding Principles that can be implemented by LHDs to make their overdose prevention programs more robust. IOPSLL recipients implemented activities in all Strategies and Guiding Principles; however, they were less likely to provide evidence-based treatment or to conduct equity-related efforts to address systematic disparities in the burden of overdose across the community. In some cases, such as MOUD, the health department was not always positioned to begin offering clinical services and CDC’s funds cannot be used for direct medical/clinical care or substance use treatment. However, their capacity to work with medical providers to enhance community awareness of treatment, improve linkages to care, and increase the level of care by educating providers about the epidemic and dispelling stigmatizing myths about their patients who use drugs may be supported. Additionally, funding requirements heavily influence certain activities being implemented. For example, the lack of evidence-based treatment interventions being implemented can be attributed to funding restrictions. LHDs were not allowed to used funds to the provision of direct medical care. Funding opportunities with fewer restrictions allow jurisdictions to use grant money to implement overdose preventions that are necessary and tailored to their local community contexts.

Other findings showed that LHDs and their partners could better support a comprehensive response to the overdose epidemic if they were able to expand or refine the scope of their services. Thus, dedicated services are warranted for subpopulations disparately impacted by the burden of overdose within communities. Some IOPSLL participant activities pointed a path forward for local efforts to better support groups at high risk of overdose. For example, some LHDs implemented programs to work with people with a history of substance use who were recently incarcerated or developed a focused communications campaign aimed at educating black, Indigenous, and people of color (BIPOC) communities on the risk of overdose and how to access support services.

Additionally, IOPSLL recipients were able to implement efforts in under-used levels of the socio-ecological model but were noticeably less likely to engage at both the relationship and policy level. Relationship-level activities, or those that directly benefit persons closely connected to those at risk of overdose such as family, friends, first responders, and healthcare providers, are important to raising the overall recovery capital for PWUD. IOPSLL recipients did perform numerous activities in this regard such as working with pharmacists or emergency medical services (EMS) to improve their individual capacity to provide services to those in need. However, data also show that the connection between health departments and the friends and families of those at risk could be deepened. A holistic response to the epidemic can only be mounted by ensuring that all PWUD’s social connections have the tools, training, information, and resources they need to support their loved ones.

Evidence-based policies can help prevent overdose, but LHDs are not always able to fully implement these policies, as they may be hinge upon federal policy and/or other types of state laws and regulations. However, an effort to elevate local voices by providing a forum or improving their internal capacity to implement policies in their communities should be considered. Additionally, increasing collaboration with other local governments and educating local elected officials about health department initiatives may be beneficial to address local regulatory barriers. Ultimately, policy discussions should be influenced by the best information on the ground, something LHDs and their partners are well equipped to provide [17].

Local jurisdictions such as cities and counties are often at the forefront of addressing a response regardless of the larger forces influencing the direction of the epidemic. LHDs need sufficient infrastructure and capacity to ensure a robust and equitable response. The lessons from CDC and NACCHO’s IOPSLL program have highlighted both the strengths local communities already exhibit and the opportunities that remain to expand and enhance their overdose prevention and response capabilities. LHDs were especially successful in implementing overdose prevention activities at the community and individual levels that aimed reduce stigma, link overdose prevention resources and wraparound services, and distribute harm reduction materials to people at risk of experiencing or witnessing an overdose. Our study also identified gaps services and identified that LHDs should provide activities related to evidence-based treatment and health equity and incorporate overdose prevention activities at the relationship and policy SEM levels.

### Limitations

The data included in our study only represent activities that were funded and supported under IOPSLL, and LHDs notably support additional overdose prevention programming. Furthermore, many of the activities implemented under IOPSLL were multidisciplinary and could have been coded under more than one HHS Overdose Prevention Strategy or Guiding Principle. Additionally, funding requirements likely impacted activities implemented and reported on by LHDs and could have influenced the observed patterns in our study results. For example, cohort 4 jurisdictions were required to embed health equity components into their work and reporting documents. For this reason, a majority of health equity activities were implemented by cohort 4 LHDs. LHDs in the other cohorts may have conducted health equity activities but did not specify them in their required reporting. These results may be an underestimate of the strategies used because activities were not double-coded. Furthermore, the currently available deliverables do not include evaluation data and thus no outcome conclusions can be made.

Additionally, a few limitations are related to the IOPSLL program composition and data collection. The request for application was not standardized between cohorts and may have influenced the activities chosen. Each IOPSLL cohort received varying degrees of TA and other project support as the program evolved from year 1 to 4. The data used to inform this paper draws from each site’s IOPSLL project workplan, which was self-reported and contained differing levels of detail from jurisdictions which led to the recategorization of some activities to fit the HHS framework. While IOPSLL sites did collect evaluation data, the process was not standardized, and the results of the evaluation are beyond the scope of this paper.

### Strengths

This study includes multiple cohorts that span across 4 years and varied geographic regions with different priority populations. Additionally, the coders and authors of this paper came from diverse backgrounds and brought unique expertise in public health and evaluation. This is the first paper to provide a descriptive landscape of overdose prevention activities LHDs implement across the nation. Our findings can be used to guide and inform newly funded Overdose Data to Action: Limiting Overdose through Collaborative Actions in Localities (OD2A: LOCAL) jurisdictions and other LHDs as they design and implement activities to prevent overdoses in their communities.

## Conclusions

This paper uses the HHS Overdose Prevention Strategies and Guiding Principles and the SEM to describe the landscape of federally funded overdose prevention activities at the local level. Localities have primarily implemented activities focused on the individual and community levels of the social ecology, with most centered around the HHS Overdose Prevention Strategies and Guiding Principles of coordination, collaboration, and integration; harm reduction; and data and evidence. Continuing these efforts is important, as is ensuring that LHDs explore opportunities to enhance and expand the scope of their overdose prevention work in various strategy areas across the social ecology.

Supporting health systems' capacity to provide evidence-based treatment, increasing equitable services to disproportionately affected populations, expanding recovery supports for PWUD, and/or driving anti-stigma efforts in various settings are all interventions that LHDs have already started to implement. However, enhancing and scaling up these efforts is possible. LHDs can also reach beyond PWUD or the community organizations that work with them: they can provide overdose prevention education and resources and engage and empower friends, families, and other persons who may directly interact with PWUD. Developing, elevating, and disseminating robust evidence-based interventions tailored for the local level will be important as federal agencies continue to directly fund localities for overdose prevention, response, and surveillance. Such interventions seize available opportunities to address the overdose epidemic through a multi-pronged approach in addition to capitalize on the strengths of LHDs.

## Data Availability

The datasets used and/or analyzed during the current study are available from the corresponding author upon reasonable request.

## Abbreviations

BIPOC: Black, indigenous, and people of color
CDC: Center for Disease Control and Prevention
CI: Coordination, collaboration, and integration
DE: Data and evidence
E: Equity
EHR: Electronic health record
EMS: Emergency medical services
ET: Evidence-based treatment
FTIR: Fourier transform infrared
HCV: Hepatitis C
HHS: United Stated Department of Health and Human Services
HIV: Human immunodeficiency virus
HR: Harm Reduction
IOPSLL: Implementing Overdose Prevention Strategies at the Local Level
LHD: Local health department
MOUD: Medications for opioid use disorder
NACCHO: National Association of City and County Health Officials
OD2A: Overdose Data to Action
OD2A: LOCAL: Overdose Data to Action: Limiting Overdose through Collaborative Actions in Localities
ODMAP: Overdose Mapping and Application Program
OUD: Opioid use disorder
PP: Primary Prevention
PWID: People who inject drugs
PWUD: People who use drugs
RS: Reducing stigma
SEM: Social-ecological model
SP: Recovery support
SUD: Substance use disorder
TA: Technical assistance

## References

[1] Ahmad FB, Cisewski JA, Rossen LM, Sutton P. Provisional drug overdose death counts. National Center for Health Statistics. 2024.

[2] Haffajee R, Sherry, TB, Dubenitz, JM, White, JO, Schwartz, D, , Stoller B, Swenson-O’Brien, AJ, Manocchio, TM, Creedon, TB, and Bagalman E. U.S. Department of Health and Human Services Overdose Prevention Strategy (Issue Brief).

[3] Krug EG, Mercy JA, Dahlberg LL, Zwi AB (2002). The world report on violence and health. Lancet.

[4] Centers for Disease Control and Prevention. The Social-Ecological Model: A Framework for Prevention. 2017;Available at: https://www.cdc.gov/violenceprevention/overview/socialecologicalmodel.html.

[5] Jalali MS, Botticelli M, Hwang RC, Koh HK, McHugh RK (2020). The opioid crisis: a contextual, social-ecological framework. Health Res Policy Syst.

[6] Overdose Data to Action CDC-RFA-CE19-1904. Grants.gov: Department of Health and Human Services Centers for Disease Control - NCIPC; 2019.

[7] National Center for Injury Prevention and Control, U.S. Department of Health and Human Services. Evidence-Based Strategies for Preventing Opioid Overdose: What’s Working in the United States. 2018.

[8] Stone EM, Kennedy-Hendricks A, Barry CL, Bachhuber MA and McGinty EE. The role of stigma in U.S. primary care physicians' treatment of opioid use disorder. Drug Alcohol Depend. 2021;221:108627. 10.1016/j.drugalcdep.2021.108627 PMC8026666 33621805

[9] Kennedy-Hendricks A, Barry CL, Gollust SE, Ensminger ME, Chisolm MS, McGinty EE (2017). Social stigma toward persons with prescription opioid use disorder: associations with public support for punitive and public health-oriented policies. Psychiatr Serv.

[10] Barry CL, McGinty EE, Pescosolido BA, Goldman HH (2014). Stigma, discrimination, treatment effectiveness, and policy: public views about drug addiction and mental illness. Psychiatr Serv.

[11] National Center for Injury Prevention and Control DoDOP. Stop Overdose. 2022;Available at: https://www.cdc.gov/stopoverdose/index.html. Accessed 9/15/23, 2023.

[12] Young LB, Grant KM, Tyler KA (2015). Community-Level Barriers to Recovery for Substance-Dependent Rural Residents. J Soc Work Pract Addict.

[13] Medina S, Van Deelen A, Tomaszewski R, Hager K, Chen N, Palombi L (2022). Relentless stigma: a qualitative analysis of a substance use recovery needs assessment. Subst Abuse.

[14] Elswick A, Fallin-Bennett A, Ashford K, Werner-Wilson R (2018). Emerging adults and recovery capital: barriers and facilitators to recovery. J Addict Nurs.

[15] Embry DD. The PAX Good Behavior Game: Schoolwide Implementation Guide. Hazelden Publishing; 2003.

[16] Jakubowski A, Fowler S, Fox AD (2023). Three decades of research in substance use disorder treatment for syringe services program participants: a scoping review of the literature. Addict Sci Clin Pract.

[17] HHS. 2023;Available at: https://www.hhs.gov/grants-contracts/grants/grants-policies-regulations/lobbying-restrictions.html, 2023.

